# Transitioning from Episodic Cardiac Missions to Sustainable National Cardiac Surgery Services in Papua New Guinea: A Mixed-Methods Programme Evaluation

**DOI:** 10.5334/aogh.5309

**Published:** 2026-05-28

**Authors:** Marco Lizwan, Ling Zhu, Noah Tapaua, Antonia Zeng, Oriana Ng, Kim Chai Chua, Yeow Leng Chua

**Affiliations:** 1 Department of Cardiothoracic Surgery, National Heart Centre Singapore, Singapore; 2 Department of Cardiothoracic Surgery, Port Moresby General Hospital, Port Moresby, Papua New Guinea; 3 Department of Anaesthesiology, Singapore General Hospital, Singapore

**Keywords:** capacity building, cardiac surgery, global surgery, health systems strengthening, implementation science, low- and middle-income countries

## Abstract

*Background:* Access to cardiac surgery remains severely limited in many low- and middle-income countries, where care is often delivered through short-term missions or overseas referrals. Papua New Guinea (PNG) is undergoing a transition towards sustainable national cardiac surgical capacity.

*Methods:* We conducted a mixed-methods evaluation of a March 2026 Singapore–PNG cardiac surgery mission at Port Moresby General Hospital. Quantitative data were collected using Likert-scale surveys assessing key domains of mission performance. Qualitative data were analysed using thematic analysis. Findings were used to identify pragmatic strategies to improve cardiac surgical delivery within existing system constraints.

*Results:* Twenty-eight participants completed the evaluation. Overall mission experience was rated highly (mean 4.61 ± 1.07). Strengths included multidisciplinary collaboration, education and increasing local leadership. However, key challenges were identified in post-operative care (protocol clarity mean 2.86 ± 1.33), ICU coordination and supply chain reliability. Workforce gaps and communication inefficiencies further impacted care delivery.

*Conclusion:* Mentorship-based cardiac missions can support local capacity development, but sustainable service delivery requires targeted improvements in post-operative care systems, workforce capability and operational processes. These findings highlight practical strategies to strengthen cardiac surgical services in resource-constrained settings.

## Introduction

Cardiovascular disease is the leading cause of global mortality, accounting for over 18 million deaths annually, disproportionately affecting low- and middle-income countries (LMICs) [1]. Despite this burden, access to cardiac surgery remains extremely limited, particularly in small island states and resource-constrained settings [2, 3].

Historically, cardiac surgical care in LMICs has relied on overseas referrals or short-term visiting missions. While such models provide immediate clinical benefit, they are inherently episodic and do not address systemic capacity gaps [4]. Increasingly, global surgery frameworks emphasise the need for integrated, locally led surgical systems to ensure equitable and sustainable access to care [5].

Papua New Guinea (PNG) exemplifies this transition. Cardiac care has historically been dependent on external missions and international referrals. However, recent developments—including partnerships with Singapore Health Services—have initiated a shift towards domestic service provision.

The Singapore–PNG Cardiac Surgery Programme was established to support this transition through service delivery, training and system strengthening. This study evaluates a recent cardiac surgery mission and identifies practical strategies to improve service delivery within existing health system constraints.

## Methods

### Study design

A mixed-methods programme evaluation was conducted during the March 2026 Singapore–PNG cardiac surgery mission at Port Moresby General Hospital. This study was conducted as a programme evaluation. Institutional approval was obtained from National Heart Centre Singapore, and the requirement for formal ethical review was waived as no patient-level data were collected and all responses were anonymised.

This study is reported in accordance with the Strengthening the Reporting of Observational Studies in Epidemiology (STROBE) guidelines [6]. Qualitative components were reported in line with recognised standards for qualitative research reporting.

### Participants

A total of 28 participants across multidisciplinary roles (surgeons, anaesthetists, perfusionists, nurses, cardiologists and physiotherapists) completed the evaluation, with representation from both Singapore and PNG teams.

### Quantitative data

Quantitative data were collected using Likert-scale questionnaires (1-5), where higher scores indicated more favourable responses. Survey domains included overall experience, pre-mission planning, intraoperative workflow, post-operative care, education and sustainability.

Descriptive statistics were used to summarise responses. Likert-scale data were treated as ordinal variables but summarised using means and standard deviations (mean ± SD) to facilitate domain-level comparison. Results were interpreted cautiously, with emphasis placed on trends rather than precise numerical differences.

No inferential statistical testing was performed, given the exploratory nature of the study and the relatively small sample size (*n* = 28). Subgroup comparisons (e.g. between Singapore and Papua New Guinea participants or across professional roles) were not conducted due to limited statistical power.

All analyses were performed using Microsoft Excel (Microsoft Corp., Redmond, WA, USA).

### Qualitative data

Open-ended responses were analysed using thematic analysis to identify key strengths, challenges and system-level issues.

## Results

### Participant characteristics

**Figure 1:**
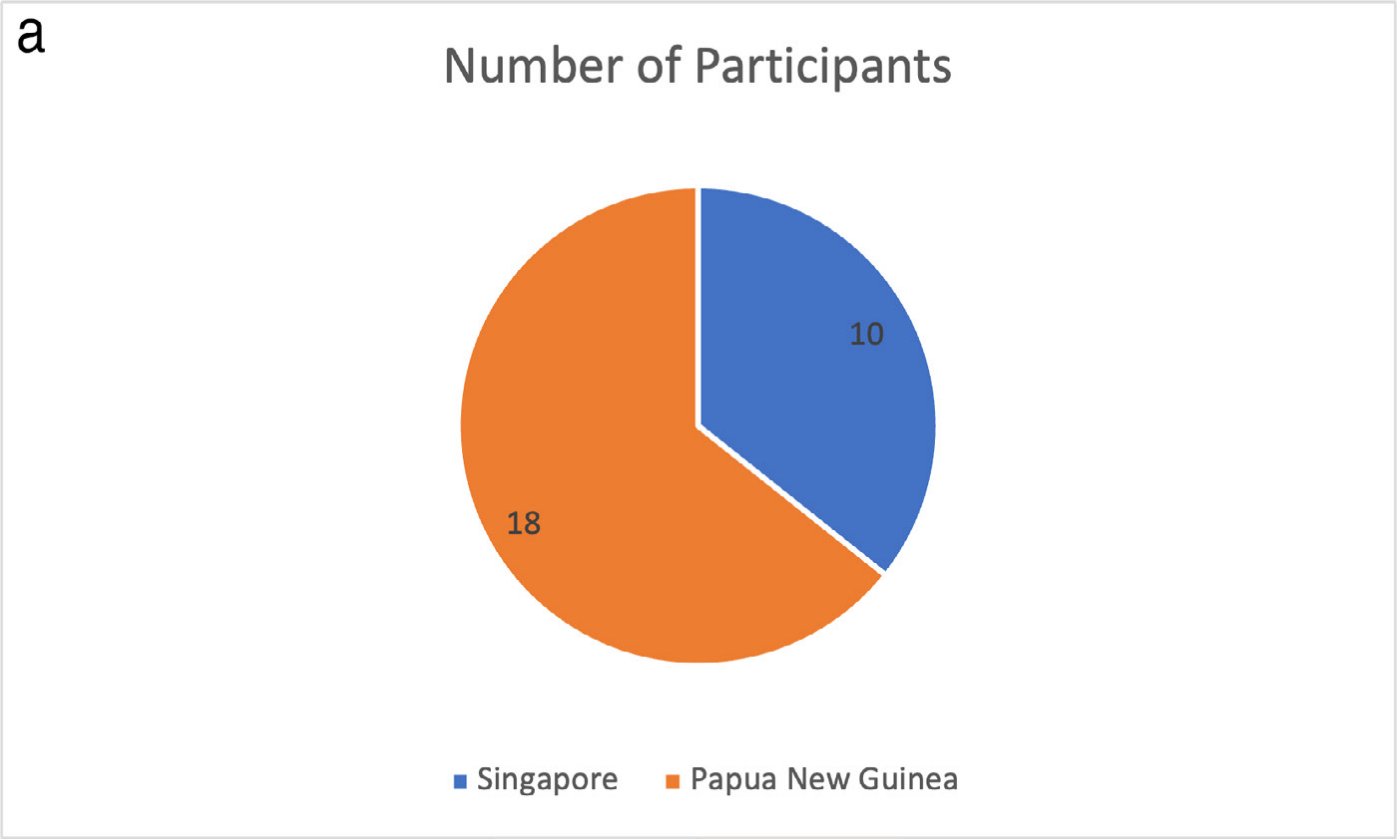

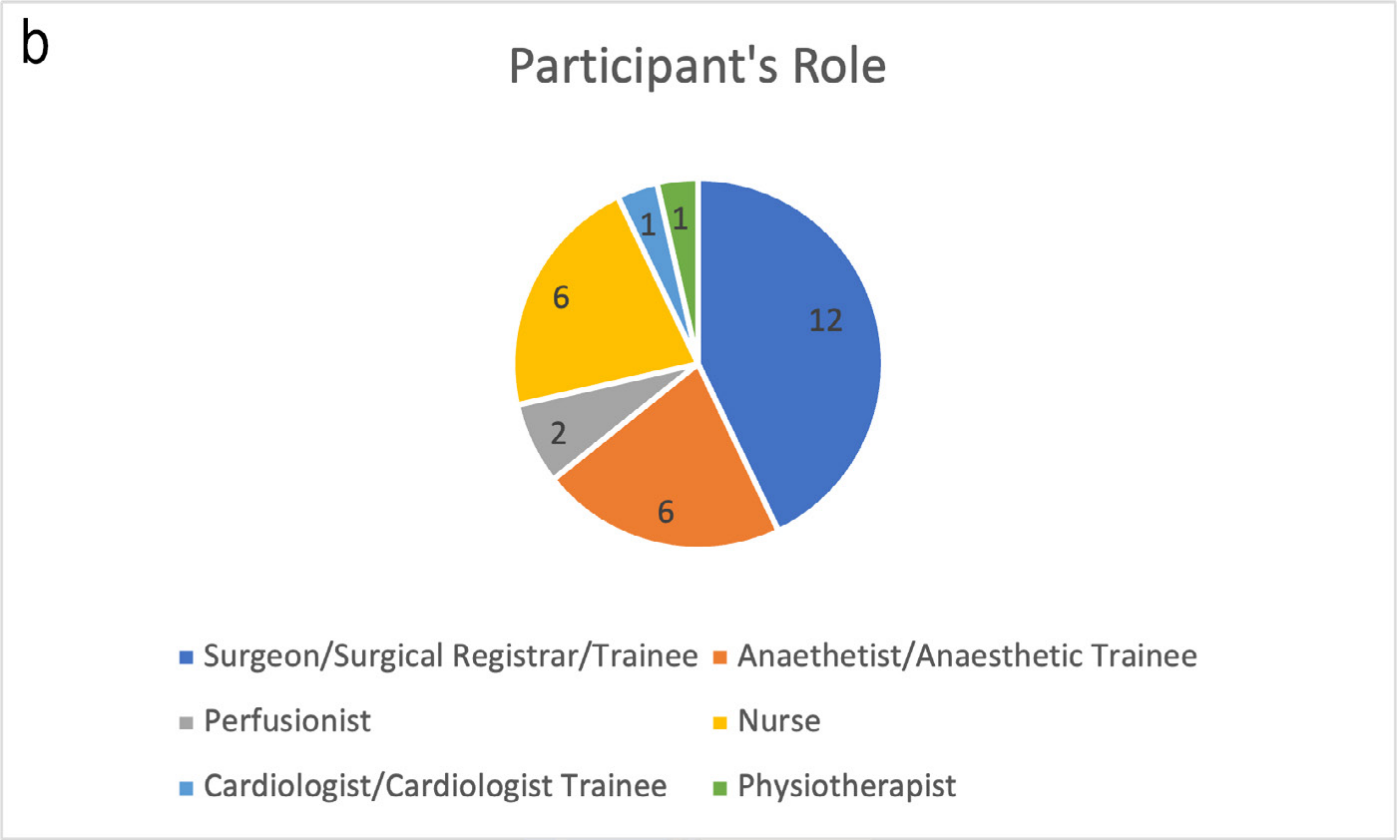
Participant characteristics by country and professional role. **(a)** Distribution of participants by country of origin. **(b)** Distribution of participants by professional role within the multidisciplinary cardiac team. Data are presented for all respondents (*n* = 28).

Of the 28 respondents, 18 were from PNG and 10 from Singapore. Participants represented a wide range of clinical roles, with the largest groups comprising surgeons (*n* = 12), anaesthesia providers (*n* = 6) and nurses (*n* = 6) (Figure 1).

### Quantitative findings

The overall quantitative findings are summarised in Table 1.

**Table 1:** Quantitative evaluation of mission domains using Likert-scale responses. Summary of participant responses to the Singapore–Papua New Guinea cardiac surgery mission evaluation (*n* = 28). Data are presented as mean ± standard deviation (SD) based on 5-point Likert-scale ratings (1 = strongly disagree/very poor, 5 = strongly agree/excellent). Domains assessed include overall experience, pre-mission planning, intraoperative workflow, post-operative care, education and capacity building, collaboration, sustainability and team well-being. Higher scores indicate more favourable perceptions.

DOMAIN	MEAN ± SD
**Overall experience**	
Objective	4.32 ± 0.94
Communication	3.86 ± 1.08
Roles and responsibilities	3.71 ± 1.01
Clinical workload	4.21 ± 0.99
Overall	4.61 ± 1.07
**Pre-mission planning and preparation**	
Adequate briefing	4.04 ± 1.14
Appropriate case selection	4.39 ± 1.03
Sufficient preoperative patient information	3.89 ± 1.17
Adequate equipment and consumable planning	3.93 ± 1.05
Well-organized travel, lodging and logistics	4.14 ± 1.04
**Intraoperative and clinical workflow**	
Efficient operating theatre flow	3.86 ± 1.11
Effective operating theatre teamwork	4.14 ± 0.97
Collaborative decision-making	4.32 ± 0.94
Patient safety standards	4.32 ± 0.98
**Post-operative care and ICU management**	
Clear post-operative care protocols	2.86 ± 1.33
Effective handover	3.68 ± 1.22
Adequate ICU staffing and resources	3.43 ± 1.29
Well-coordinated complication management	3.46 ± 1.07
**Education, skills transfer and capacity building**	
Adequate teaching and learning opportunities	4.14 ± 0.93
Bi-directional knowledge exchange	4.04 ± 1.07
**Cultural and professional collaboration**	
Collegial and respectful working relationship	4.43 ± 0.92
**Sustainability and long-term Impact**	
Mission aligns with long-term PNG goals	4.57 ± 0.96
Sustainable system locally	3.86 ± 1.01
Realistic and achievable follow-up plan	3.96 ± 1.07
**Well-being and team support**	
Manageable workload	4.18 ± 1.09
Adequate rest and recovery	3.68 ± 1.19
Sufficient emotional and psychological support	3.89 ± 1.26

Pre-mission planning domains were generally well rated, including case selection (4.39 ± 1.03) and logistics (4.14 ± 1.04). However, patient information (3.89 ± 1.17) and equipment planning (3.93 ± 1.05) scored lower. Intraoperative performance was rated positively, particularly for collaborative decision-making (4.32 ± 0.94) and patient safety (4.32 ± 0.98), although operating theatre flow was less consistent (3.86 ± 1.11). Post-operative care domains were consistently lower, particularly protocol clarity (2.86 ± 1.33), intensive care unit (ICU) staffing (3.43 ± 1.29) and complication management (3.46 ± 1.07). Education and collaboration domains were highly rated, including teaching opportunities (4.14 ± 0.93) and collegiality (4.43 ± 0.92). Participants also reported strong alignment with long-term goals (4.57 ± 0.96), although perceptions of sustainability were more moderate (3.86 ± 1.01).

### Qualitative findings

#### Key strengths

The mission was characterised by strong multidisciplinary collaboration and shared ownership of patient care across all phases. Participants consistently described cohesive teamwork between Singapore and PNG clinicians, enabling effective coordination from preoperative planning to intraoperative execution and post-operative management.

Education and knowledge transfer emerged as a central strength. Structured teaching sessions, intraoperative mentoring and bedside ICU discussions facilitated meaningful capacity building. Participants reported gaining practical skills in cardiac surgery, echocardiography, perioperative management and critical care.

Importantly, the mission demonstrated increasing local clinical leadership. PNG clinicians were actively involved in surgical procedures and perioperative decision-making, reflecting a shift from passive participation towards independent practice. This transition represents a critical step towards sustainable national cardiac services.

#### Key operational challenges

The most significant challenges were observed in post-operative care. Participants highlighted a lack of standardised ICU protocols, unclear haemodynamic targets and inconsistent escalation pathways. These gaps contributed to uncertainty in managing complications such as arrhythmias, haemodynamic instability and pacing requirements.

Communication gaps were also evident, particularly during handovers and in defining roles and responsibilities. These issues were compounded by variability in documentation practices and limited continuity of care during off-hours. In addition, inconsistencies in patient listing and resource allocation—such as sequencing of cases and access to echocardiography—led to inefficiencies during workflow.

Resource constraints further impacted workflow. Limited availability and standardisation of equipment, inconsistent consumable supply and infrastructure limitations created inefficiencies in both intraoperative and post-operative settings.

Workforce limitations were also evident, particularly among non-physician cadres. Participants highlighted gaps in specialised nursing care, perfusion support and physiotherapy, particularly in the post-operative setting. Limited familiarity with cardiac-specific monitoring, escalation protocols and rehabilitation pathways contributed to variability in care delivery. These gaps were compounded by the absence of structured training pathways for non-physician staff.

## Discussion

This study highlights both the potential and limitations of mentorship-based cardiac surgery missions in LMIC settings.

The high ratings for collaboration, education and mission impact demonstrate that mentorship-based mission models can effectively support skill transfer and local capacity development. These findings align with previous studies showing that structured partnerships can accelerate surgical training in resource-limited settings [7, 8].

However, the consistently lower scores in post-operative care domains underscore a critical bottleneck. Cardiac surgery is uniquely dependent on robust ICU systems, and deficiencies in post-operative care represent a key bottleneck in programme sustainability.

The findings also reinforce the importance of health system strengthening. According to the Lancet Commission on Global Surgery, sustainable surgical systems require coordinated development across infrastructure, workforce, service delivery and governance [5]. The challenges identified in this study—particularly in ICU care, supply chains and workforce—reflect systemic limitations rather than technical deficits.

An important finding from this study is the critical role of non-physician staff in sustaining cardiac surgical services. While physician training is often prioritised in mission-based programmes, the effectiveness of cardiac surgery is highly dependent on specialised nursing, perfusion and allied health support. Deficiencies in these areas—particularly in post-operative ICU care—represent a key constraint to programme sustainability.

### Strengthening non-physician workforce development

Addressing this gap requires a structured and programmatic approach to non-physician education.

Pre-mission education should be expanded to include targeted training for nursing and allied health staff, focusing on core competencies such as haemodynamic monitoring, post-operative pathways and early recognition of complications. Delivering such training prior to missions may improve baseline readiness. During missions, bedside teaching should be formalised with clearly defined learning objectives. Training should be aligned with task-specific competencies, enabling staff to develop practical skills relevant to their roles. Post-mission continuity is equally important. Virtual teaching sessions, case discussions and shared educational materials may help sustain learning between missions.

Standardised protocols and checklists can further support non-physician staff by reducing variability and providing clear guidance for routine cases. Finally, future missions should incorporate dedicated non-physician educators, such as senior ICU nurses and perfusion trainers, to facilitate targeted capacity building.

### Pragmatic strategies to improve service delivery

Rather than proposing large-scale system transformation, our findings highlight practical strategies that can be implemented within existing constraints.

First, strengthening post-operative care pathways is critical. The low rating for protocol clarity underscores the need for standardised ICU management frameworks. Practical interventions include the development of locally adapted post-operative protocols, structured ICU handover templates and pre-mission education sessions to align expectations regarding post-operative care.

Second, communication processes can be improved through relatively simple interventions. Expanding multidisciplinary discussions to include all hands-on team members—including ICU nurses and junior staff—may enhance shared understanding of patient management plans. The use of structured communication tools, such as standardised handover formats and shared messaging platforms (e.g. WhatsApp groups), may further improve coordination and responsiveness to clinical changes.

Third, workflow efficiency may be improved through better preoperative planning and clearer allocation of resources. Early confirmation of patient lists, improved coordination of equipment availability (e.g. echocardiography machines), and structured preoperative briefings involving all relevant disciplines may reduce intraoperative delays and improve utilisation of limited resources.

Fourth, resource constraints should be explicitly incorporated into planning processes. Limited ICU capacity, for example, should inform patient selection and scheduling. While supply chain limitations may not be immediately modifiable, improved pre-mission inventory planning and communication can mitigate disruptions.

Finally, addressing workforce limitations requires targeted and context-sensitive approaches. Short-term strategies may include expanding the composition of visiting teams to include key specialities such as intensivists, cardiologists and physiotherapists. In parallel, mission design should account for local manpower constraints, ensuring that care delivery and teaching activities are aligned with available staffing capacity.

A summary of identified challenges and corresponding pragmatic solutions is provided in the supplementary material (Table 1).

## Conclusion

Papua New Guinea is at a pivotal stage in developing sustainable cardiac surgical services. While mentorship-based missions can catalyse capacity building, long-term success depends on addressing systemic constraints in the workforce, infrastructure and governance.

The PNG experience provides an important model for other LMICs seeking to transition from episodic missions to sustainable national surgical systems. In particular, strengthening non-physician workforce capacity is essential to ensuring consistent and safe delivery of cardiac surgical care. Pragmatic, context-specific improvements may offer a feasible pathway towards sustainable service development in resource-limited settings.

## Key Questions

### What is already known on this topic

- Cardiac surgery access in LMICs is extremely limited and often dependent on short-term missions.
- Episodic mission models provide immediate care but rarely achieve long-term sustainability.

### What this study adds

- Demonstrates how a mentorship-based cardiac mission can transition towards local clinical ownership.
- Identifies post-operative care, supply chain and workforce gaps as primary system bottlenecks.
- Provides a pragmatic, system-level strategies to improve cardiac surgical delivery within constrained settings

### How this study might affect research, practice or policy

- Supports policy shifts from mission-based care towards system-oriented service delivery models.
- Highlights actionable improvements in ICU care, workflow and communication.
- Offers transferable insights for LMICs developing cardiac surgical capacity.

## Data Availability

Additional data are available from the corresponding author on reasonable request.

## Additional File

The additional file for this article can be found as follows:

Supplementary Table 1. Identified operational challenges and proposed pragmatic solutions.
