# Supplementary material for: Transitioning from Episodic Cardiac Missions to Sustainable National Cardiac Surgery Services in Papua New Guinea: A Mixed-Methods Programme Evaluation
Source: Ann Glob Health. 2026 May 28;92(1):50. doi: 10.5334/aogh.5309 (PMC13220737; doi:10.5334/aogh.5309)
Supplement: Supplementary Table 1. — Identified operational challenges and proposed pragmatic solutions. [file agh-92-1-5309-s1.pdf]

Supplementary Table 1. Identified operational challenges and proposed pragmatic solutions

Domain	Identified Problem	Proposed Solution
Communication	Limited involvement of ICU nurses and junior staff in multidisciplinary discussions	- Expand meeting attendance<br>- Circulate minutes to all team members
	Confusion regarding patient listing and resource allocation (e.g., case sequencing, echocardiography access)	- Improve preoperative coordination<br>- Establish centralised communication channels
	Unclear postoperative plans and expectations	- Pre-mission education<br>- Develop postoperative protocols and ICU handover templates
	Delayed updates and lack of real-time coordination	Use shared messaging platforms (e.g., WhatsApp groups)
Resource Constraints	Limited ICU bed capacity	Incorporate ICU capacity into patient selection and surgical scheduling
	Variability in consumables availability	Improve pre-mission inventory planning and communication

Workforce	Shortage of specialised roles (intensivists, perfusionists, physiotherapists)	Expand visiting team composition to include key specialties where feasible
	Limited manpower affecting care delivery and training	- Align mission scope with available staffing<br>- Incorporate manpower planning into programme design
